# Comparison of Histological, Clinical, and Radiographic Outcomes of Postextraction Ridge Preservation by Allogenic Bone Grafting With and Without Injectable Platelet-Rich Fibrin: A Double-Blinded Randomized Controlled Clinical Trial

**DOI:** 10.1155/2024/8850664

**Published:** 2024-10-24

**Authors:** Mohammad Reza Talebi Ardakani, Zeinab Rezaei Esfahrood, Fatemeh Mashhadiabbas, Masoud Hatami

**Affiliations:** ^1^Department of Periodontics, School of Dentistry, Shahid Beheshti University of Medical Sciences, Tehran, Iran; ^2^Department of Oral and Maxillofacial Pathology, School of Dentistry, Shahid Beheshti University of Medical Sciences, Tehran, Iran; ^3^Department of Periodontics, School of Dentistry, Kermanshah University of Medical Sciences, Kermanshah, Iran

**Keywords:** allografts, alveolar ridge preservation, bone regeneration, platelet-rich fibrin, tooth socket

## Abstract

**Objectives:** This randomized controlled clinical trial compared the histological, clinical, and radiographic outcomes of postextraction ridge preservation by allogenic bone grafting with and without injectable platelet-rich fibrin (I-PRF).

**Materials and Methods:** Twenty single-rooted maxillary and mandibular teeth to be extracted and replaced by dental implants were randomly divided into two groups (*n* = 10). Cone-beam computed tomography (CBCT) scans were obtained preoperatively to assess bone dimensions and ridge width. The teeth were then extracted, and tooth socket preservation was performed with allograft and collagen type 1 in the control group and allograft, collagen type 1, and I-PRF in the intervention group. CBCT scans were obtained again 3 months after the first stage of surgery, and the second stage of surgery was performed for implant placement, ridge width measurement, and obtaining a biopsy sample. Radiographic bone width, clinical bone width, and radiographic bone height were measured. A histomorphometric method was applied to quantify residual graft material, new bone formation, and nonmineralized tissues. The data were analyzed with Student's *t*-test and Mann–Whitney *U* test (*α* = 0.05).

**Results:** The intervention group showed a significantly smaller reduction in radiographic bone width (*P*=0.038) and clinical bone width (*P*=0.033), reduction in radiographic bone height (*P*=0.213) was not significant. A significantly lower percentage of residual graft particles (*P*=0.021) and a significantly higher mean percentage of newly formed bone (*P*=0.038) than the control group. However, the difference in the percentage of nonmineralized tissue (*P*=0.208) was not significant.

**Conclusion:** Despite the optimal outcome of ridge preservation in both groups, the application of allograft plus I-PRF yielded superior histological, clinical, and radiographic results compared with allograft alone, and this difference was significant in most variables.

## 1. Introduction

Tooth extraction is a common dental procedure that may be indicated due to trauma, endodontic treatment failure, root fracture, extensive irreparable caries, and hopeless periodontal prognosis [1]. As gerodontology is a new discipline of modern dentistry that deals with all the oral problems of patients over the age of 65, and implantation in extracted sites is one of them [2]. Physiological remodeling of alveolar bone takes place following tooth extraction and results in a reduction in ridge dimensions, its deformation, and eventual bone loss [3]. It has been reported that the alveolar bone width often undergoes a 50% reduction in the first year after tooth extraction, decreasing from 12 to 5.9 mm on average, and two-thirds of this change occurs in the first 3 months after extraction. The percentage reduction was somewhat larger in the molar regions than in the premolar regions and in the mandible compared with the maxilla [4].

Bone resorption following tooth extraction compromises dental implants' ideal positioning and success [5]. Therefore, preservation of the alveolar ridge following tooth extraction is necessary for optimal placement of dental implants and prosthetic reconstruction [6]. Thus, researchers are searching for new strategies for socket preservation to preserve the alveolar ridge, prevent bone resorption, and subsequently maximize the success of dental implant treatment [7]. In case of poor socket preservation, microsurgical access flap techniques [8] and bone grafting may be required for bone regeneration, particularly in the buccal surface, to improve the outcome of dental implant treatment [6].

Several strategies may be adopted for socket preservation to enable ideal implant placement, such as the application of autografts, xenografts, allografts, alloplastic materials, mesenchymal stem cells, and bone morphogenetic proteins [9]. In a review study, Jambhekar, Kernen, and Bidra [10] reported that xenografts had the highest efficacy for socket preservation, followed by allografts and alloplastic materials, compared with the natural healing of the alveolar ridge. Despite the advantages of the aforementioned graft materials, they may not yield favorable results in all cases due to their avascular nature [11]. Moreover, after application, high amounts of residual graft material may compromise and decrease the bone-to-implant contact [12]. Maxillary sinus floor augmentation with allograft alone compared with alternate grafting material may show the possible histologic differences between grafting materials [13].

Platelet-rich fibrin (PRF) was introduced in 2001 and has been extensively used since then [14]. The application of PRF for tooth socket preservation [15], treatment of gingival recession defects [16–18], regeneration of intrabony periodontal defects, and clinical management of oral mucositis to improve the life quality of patients' life [19] and maxillary lateral and mini crestal sinus floor elevation [20] has been associated with favorable results. PRF, a platelet concentrate, comprises an autologous dense fibrin matrix and growth factors certainly trapped within the fibrin meshes and slowly released, which might enhance soft and hard tissue regeneration [21–23]. Evidence shows that PRF is a rich source of growth factors, including transforming growth factor beta-1, vascular endothelial growth factor, and platelet-derived growth factor [23, 24]. These growth factors are entrapped in the PRF fibrin matrix; thus, they have a slow, gradual, and sustained release profile through the clot's natural maturation and reorganization [22, 23].

Recent advances in preparing PRF have led to the development of advanced PRF (A-PRF), which enables the release of higher amounts of growth factors due to using a lower g-force compared with PRF [25, 26]. In other words, the centrifugation speed is decreased to 1500 rpm while the duration of centrifugation increases to 14 min to prepare A-PRF. It has been reported that B and T lymphocytes are better entrapped in the matrix, and neutrophils and platelets would be more evenly distributed in this protocol [25].

The injectable form of PRF (I-PRF) is a simple liquid form of platelet concentrate that can be used alone or in combination with other biomaterials. Its preparation requires a slower centrifugation speed (i.e., 700 rpm [60 g]) and a shorter centrifugation time (i.e., 3 min). It has more regenerative cells with higher concentrations of growth factors compared with other formulations of PRF that require a higher centrifugation speed [27, 28].

Given the points that were previously addressed, this study compared the histological, clinical, and radiographic outcomes of postextraction ridge preservation by allogenic bone grafting with and without I-PRF. The null hypothesis of the study was that the histological, clinical, and radiographic outcomes of postextraction ridge preservation would not be significantly different by applying allogenic bone grafting with and without I-PRF.

## 2. Materials and Methods

This study was conducted at the Periodontics Department of the School of Dentistry, Shahid Beheshti University of Medical Sciences, Tehran, Iran, between October 2022 and March 2023. The study protocol was approved by the Ethics Committee of the University (IR.SBMU.DRC.REC.1401.066) and registered in the Iranian Registry of Clinical Trials (IRCT20221227056937N1).

### 2.1. Trial Design

In this parallel randomized controlled clinical trial, the control group underwent socket preservation by allograft alone while the intervention group underwent socket preservation by allograft plus I-PRF. The trial was reported under the guidelines of the Consolidated Standards of Reporting Trials (CONSORT).

### 2.2. Participants, Eligibility Criteria, and Settings

The inclusion criteria consisted of age >18, single-rooted teeth to be extracted and replaced by dental implants, available bone support >50% of root length after tooth extraction, systemic health of the patients (The American Society of Anesthesiologists (ASA) I and II), and absence of acute infection or extensive periapical lesion.

The exclusion criteria were systemic diseases affecting bone healing, intake of medications affecting bone healing, pregnancy, smoking >10 cigarets/day, history of radiotherapy, contraindications for implant placement, having a prosthesis at the site, requiring antibiotic prophylaxis, and presence of acute infection or extensive periapical lesion at the site of extraction.

The sample consisted of 12 eligible patients who required the extraction of maxillary or mandibular single-rooted teeth and replacement of the teeth with dental implants, presenting to the School of Dentistry of Shahid Beheshti University of Medical Sciences.

### 2.3. Interventions

Written informed consent was obtained from all patients before treatment and study enrollment.

**Figure 1:**
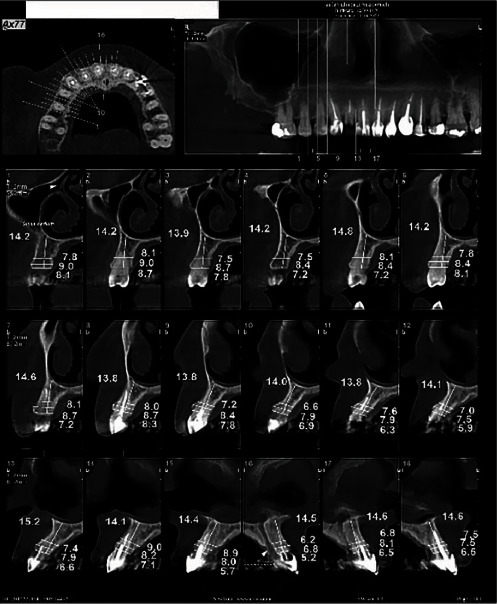
Preoperative CBCT scan of a patient. CBCT, cone-beam computed tomography.

The patients initially underwent cone-beam computed tomography (CBCT) of the respective site(s) to assess bone dimensions (Figure 1). To ensure the reproducibility of the measurements, radiographic sections were made parallel to the longitudinal axis of an adjacent tooth (which served as a reference), and the slice thickness was adjusted at 1 mm in both preoperative and postoperative CBCT scans. The section passing through the apex of the respective tooth was selected as the reference section, and the respective measurement site was identified by counting the number of slices in the interval between the reference section and the respective measurement site. Bone width was measured 3 mm below the bone crest, and bone height was measured as the distance between the bone crest and an anatomic landmark.

Prior to the intervention, an alginate impression was made from the respective jaw and poured with dental stone to fabricate an acrylic surgical stent to precisely identify the previous root location for biopsy sampling.

#### 2.3.1. Tooth Extraction

The patients received oral hygiene instructions (correct toothbrushing and flossing) before surgery. Also, they underwent scaling and root planing by manual curettes and ultrasonic instruments, if required. The patients prophylactically received 1 g of amoxicillin orally 1 h prior to tooth extraction and used 0.2% chlorhexidine (CHX) mouthwash before surgery. Local anesthesia was achieved with a 2% lidocaine injection. An intrasulcular incision was made, and the respective tooth was moved by elevator and removed by forceps atraumatically. The extraction socket was debrided with a curette to remove granulation tissue and was then rinsed with saline.

#### 2.3.2. Surgical Protocol

Twenty extraction sockets (more than one in some patients) of maxillary and mandibular single-rooted teeth were randomly assigned to two groups as follows.

##### 2.3.2.1. Control Group

Allograft material (Itp @Regen) with 500‒1000 µm fine granules + collagen type 1 (Botiss Dental GmbH, Germany).

##### 2.3.2.2. Intervention Group

Allograft material (Itp @Regen) with 500‒1000 µm fine granules + I-PRF + collagen type 1 (Botiss Dental GmbH, Germany).

In the control group, after administering local anesthesia, a mucoperiosteal flap was elevated by minimal soft tissue retraction to minimize crestal bone resorption. For reproducible clinical measurement of ridge width by measuring a certain distance from the proximal cementoenamel junction of the adjacent tooth (reference tooth), a fixed point was considered the measurement site in the sagittal plane. At this point, 3 mm apical to the ridge crest, ridge width was measured by a digital caliper (Fowler, USA) before tooth extraction and recorded. Soft tissue was taken into account in this measurement. However, by probing certain areas, the thickness of the buccal and lingual/palatal soft tissue was determined and subtracted from the final size of the ridge width to determine the alveolar ridge's buccolingual dimension. The tooth was then extracted atraumatically by the flap-less technique with a Periotome (Devemed GmbH, Germany). Allograft material was prepared as instructed by the manufacturer, and the socket was filled with a fine-grain (500‒1000 µm) allograft material, covered with collagen type 1, and sutured by 4–0 resorbable vicryl sutures (Ethicon, USA) using the figure of eight suturing technique.

In the intervention group, ridge dimensions were measured, and tooth extraction was carried out, as explained for the control group. Also, 10 mL of antecubital venous blood was collected by a 21-gauge needle, transferred into a test tube with no anticoagulant, and centrifuged at 700 rpm for 3 min. For the preparation of liquid PRF, we need to delay coagulation so that the PRF stays liquid long enough for us to be able to inject it. For this, the best tubes are additive-free orange-top polyethylene terephthalate (PET) plastic vacutainers. PET plastic is hydrophobic in nature and repels water, and hence the platelets. This prevents the activation of platelets during centrifugation and delays the start of clot formation by 15–20 min. This time is enough to collect the liquid PRF and inject it. Unlike PRF clot preparation, the top of the vacutainer should not be opened during the preparation and while withdrawing liquid PRF. Exposure to air may lead to the initiation of clotting. This centrifuge has a fixed angle with a radius of 110 mm. The upper 1 mL of the preparation layer was then removed using an 18 g 1.5-inch blunt fill needle into a 1 mL syringe. The absence of anticoagulant in the tube allowed for the formation of a fibrin clot between the red blood cells at the bottom and acellular plasma at the top. I-PRF remains in liquid form for only 15 min. Thus, it was immediately used. Allograft was prepared as instructed by the manufacturer, mixed with I-PRF, and the socket was filled with the mixture, covered with collagen type 1, and sutured with 4–0 resorbable vicryl sutures using the figure of eight suturing technique.

### 2.4. Second-Stage Surgery, Biopsy, and Implant Placement

**Figure 2:**
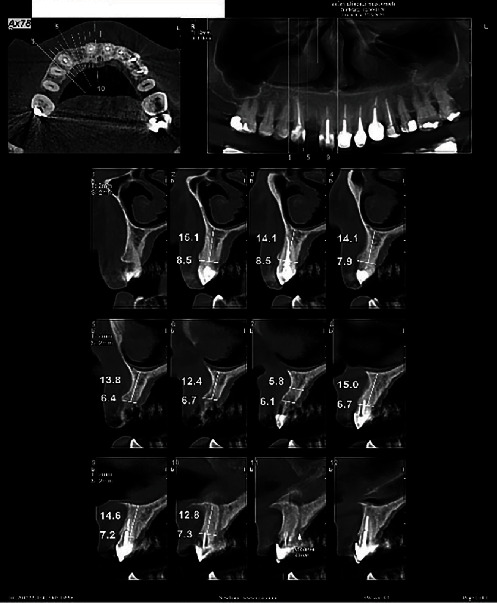
Postoperative CBCT scan of a patient. CBCT, cone-beam computed tomography.

**Figure 3:**
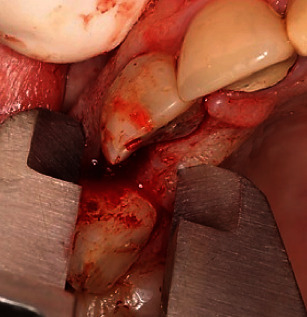
Measuring the alveolar bone width with a digital caliper.

Three months after the first-stage surgery, the patients were recalled for the second-stage surgery. A CBCT scan was obtained from the site, and the reference section was selected, as explained earlier (Figure 2). Bone dimensions were also measured. After administering local anesthesia and elevating a full-thickness mucoperiosteal flap, the respective site was identified, as explained earlier, and the ridge width was measured by a digital caliper (Figure 3). To identify the desired site for biopsy, a surgical stent was fabricated with light-cured acrylic resin on the patient's cast prepared earlier. A Trephine bur (Meisinger, Germany) was used to collect a biopsy sample measuring 2 × 4 mm from the respective site after intraoral placement of the stent. The biopsy sample was fixed in 10% formalin for 24 h and sent to a pathology laboratory for histological and histomorphometric analyses. The dental implant was then placed according to the standard protocol.

### 2.5. Histological and Histomorphometric Assessments

The biopsy samples were stored in 10% formalin for at least 5 days and decalcified in 10% formic acid for 3 days. They were rinsed with water and placed back in formalin. After 24 h, the samples were embedded in paraffin blocks, sectioned into 3-µm slices apicocoronally by a microtome, and underwent staining with hematoxylin and eosin (H&E). From each specimen, a minimum of three slices were prepared at the largest height and diameter and underwent histological and histomorphometric analyses by an experienced oral pathologist blinded to the group allocation of the samples. For histomorphometric assessments, images were taken from the entire section by a digital camera (E8400; Nikon, Japan) under a microscope (E400 Eclipse; Nikon, Japan) at ×40 magnification, and the percentages of newly formed bone, residual graft materials, and connective tissue (and other nonmineralized tissues) were calculated as the percentage of occupied area from the entire surface area of the specimen. Histological assessments were performed at ×40, ×100, and ×400 magnifications to determine the newly formed bone's volume around the graft material and immunological reactions around the graft particles. The inflammation type and degree and presence of osteoclasts and connective tissue were recorded.

### 2.6. Primary and Secondary Outcomes

The primary aim of this study was to compare the histological, clinical, and radiographic outcomes of postextraction ridge preservation by allogenic bone grafting with and without I-PRF.

### 2.7. Sample Size

The sample size was calculated at *n* = 10 in each group (20 in total) according to a previous study [29] at *α* = 0.05, *β* = 0.2, a study power of 80%, 2.2 and 1.5 mm of mean reduction in radiographic bone width, and standard deviation values of 0.6 and 0.4 in the control and test groups, respectively.

### 2.8. Interim Analyses and Stopping Guidelines

There were no interim analyses and stopping guidelines in the present study.

### 2.9. Randomization

Group allocation was random in the present study (*n* = 10) using a random allocation software program. Random Allocation Software is a program created in Microsoft Visual Basic 6, and it installs in the same way as ordinary Windows software (i.e., running setup.exe and following on-screen instructions). Once installed and run, there are some controls in the main window for specifying the number of groups (2–16), sample size, and the name of each group. It also contains menu items to determine the program output and randomization settings. The default program output is saved into either html or text files, and it may also have output to a window or to the system clipboard. A variety of randomization options can be set in the options window. The length of generated UIs (named as Code in the program) can be between 3 and 10 characters, and there are options for different alphanumeric structures. During execution, the program produces a random sequence of allocation using the Rnd function that generates a floating point random number. The Rnd function uses the linear-congruent method for pseudo-random number generation. Dark, coded envelopes were then used for random allocation concealment. Randomization was done once for the type of surgery before starting the operation and once for the sample number that was selected.

### 2.10. Blinding

The pathologist performing histological and histomorphometric assessments and the statistician analyzing the data were blinded to the samples' group allocation.

### 2.11. Statistical Analysis

The measures of central dispersion were reported for decreases in radiographic bone height and width, reduction in clinical width, and percentages of newly formed bone, residual graft particles, and nonmineralized tissues. The Shapiro–Wilk test revealed the normal distribution of all data (*P*  > 0.05). Thus, Student's *t*-test was used to compare the two groups regarding the variables. All the statistical analyses were carried out using SPSS 25 (SPSS Inc., IL, USA) at a 0.05 significance level.

Student's *t*-test needed various preconditions as follows:

• The data are continuous;
• The sample data have been randomly sampled from a population;
• There is homogeneity of variance (i.e., the variability of the data in each group is similar);
• The distribution is approximately normal.

## 3. Results

### 3.1. Participant Flow

**Figure 4:**
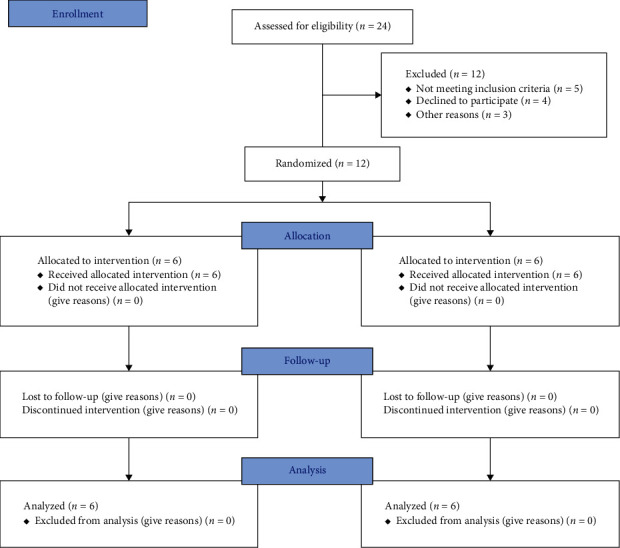
CONSORT flow diagram of patient selection and allocation. CONSORT, Consolidated Standards of Reporting Trials.

The samples consisted of 20 extraction sockets in 12 patients (7 females and 5 males) with a mean age of 42 ± 18 years. All the patients completed the treatment successfully, with no dropouts (Figure 4). The time interval between tooth extraction and dental implant placement was an average of 97 ± 25.3 days in the control group and 92 ± 1.2 days in the intervention group, with no significant difference (*P*  > 0.05). Optimal healing occurred uneventfully in both groups with no complications in any patient.

### 3.2. Harms

No harm was inflicted on the patients during the study.

### 3.3. Subgroup Analyses

#### 3.3.1. Primary Outcome

**Figure 5:**
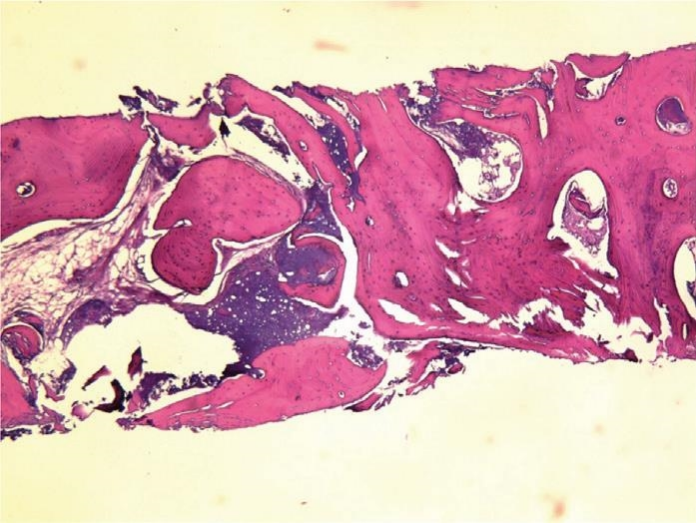
Histological view of a specimen in the intervention group at ×40 magnification. Allograft particles are surrounded by woven bone.

**Figure 6:**
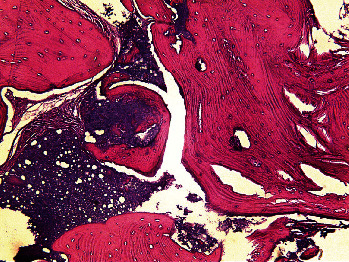
Histological view of a specimen in the intervention group at ×100 magnification.

**Figure 7:**
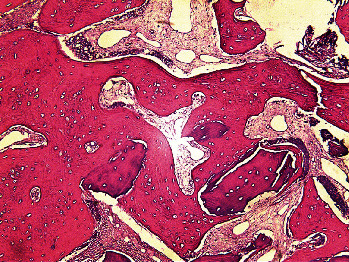
Histological view of a specimen in the control group at ×100 magnification.

Table 1 presents the measures of central dispersion for the variables measured in the two groups. As shown, the intervention group showed a significantly smaller reduction in radiographic bone width (*P*=0.038) and clinical bone width (*P*=0.033), a significantly lower percentage of residual graft material (*P*=0.021), and a significantly higher mean percentage of newly formed bone (*P*=0.038) compared with the control group. However, the two groups had no significant differences in the percentage of nonmineralized tissue (*P*=0.208) and reduction in radiographic bone height (*P*=0.213). Figures 5–7 show histological views of the intervention and control groups. The specimens showed no inflammation, bleeding, or foreign body reactions on their histological sections.

## 4. Discussion

This study compared the histological, clinical, and radiographic outcomes of postextraction ridge preservation by allogenic bone grafting with and without I-PRF. The null hypothesis of the study was that the histological, clinical, and radiographic outcomes of postextraction ridge preservation would not be significantly different using allogenic bone grafting with and without I-PRF. The results generally showed the superiority of allograft + I-PRF compared with allograft alone for socket preservation such that the intervention group showed a significantly smaller reduction in radiographic bone width (*P*=0.038) and clinical bone width (*P*=0.033), a significantly lower percentage of residual graft material (*P*=0.021), and a significantly higher mean percentage of newly formed bone (*P*=0.038) compared with the control group. Thus, the study's null hypothesis was generally rejected (except for the difference in the percentage of nonmineralized tissue and reduction in radiographic bone height).

PRF is a nonhomogenous biomaterial in which the cells are seeded. It contains different types of cytokines, leukocytes, and proteins entrapped in a highly dense fibrin membrane. This fibrin matrix has significant effects on osteoblastic differentiation [30]. The main mechanism of action of PRF is through its fibrin matrix, in which the platelets are entrapped and probably release cytokines after a certain period [31]. Thus, it appears that the dense fibrin matrix of PRF plays a role in enhancing wound healing. Also, PRF enables sustained and controlled release of growth factors over time [32].

According to the results of the present study, the difference in the percentage of nonmineralized tissue (*P*=0.208) and reduction in radiographic bone height (*P*=0.213) was not significant, although the results were slightly more favorable in the intervention group, which might be of clinical significance. A higher rate of osteogenesis in the intervention group might be related to internal growth factors and concentration in the I-PRF such that growth factors bind to each other within the fibrin matrix. After reorganization, the fibrin enables sustained release of growth factors at proper times to induce bone regeneration.

Clark et al. [33] reported a minimum ridge width and height reduction in the A-PRF + freeze-dried bone allograft (FDBA) group. In their study, the intervention group was superior to the control group in all the parameters related to ridge preservation, and the majority of differences were statistically significant. The present results were consistent with their findings, although they used A-PRF instead of I-PRF. Many previous studies assessed the efficacy of PRF application in different ridge preservation techniques [34, 35]. A previous study [35] reported superior alveolar ridge preservation with PRF compared with blood clots alone. Thakkar et al. [34] demonstrated that ridge preservation using PRF and demineralized FDBA (DFDBA) gave rise to less dimensional changes in the ridge compared to treatment with DFDBA alone. They found a significantly smaller reduction in ridge width in the case group; however, the difference in ridge height between the two groups was not significant at different time intervals. Their findings somehow coincided with the present study results, although DFDBA was not used in the present study.

Changes in ridge dimensions in the two groups in the current study coincided with a systematic review of different ridge preservation techniques using mineralized bone substitutes [36]. Girish Kumar et al. [37] investigated the efficacy of PRF as a bone substitute for alveolar ridge preservation after tooth extraction, reporting that despite the positive efficacy of PRF in the enhancement of healing and reduction of alveolar bone loss (height and width), it was not significantly different from other modalities and the control group (with no graft material). Their findings regarding no effect of PRF on reducing ridge height were in line with the present findings; however, their results regarding other variables were different from the current results, which can be due to the type of PRF or graft material used in the two studies. According to Zhang et al. [38], there were no significant differences in the mean ridge height, palatal/lingual ridge height, and ridge width between the PRF and control groups. Similarly, Suttapreyasri and Leepong [39] reported no significant differences between the PRF and control groups in ridge preservation and showed insignificant efficacy of PRF in enhancing soft tissue healing in the first 4 weeks after the intervention. Discrepancies between their findings and the present study may be attributed to using PRF with different protocols.

Histological and histomorphometric analyses in the present study revealed a significantly higher percentage of osteogenesis and a significantly lower percentage of residual graft particles in the PRF group. The type of connective tissue was loose in the PRF group and mostly fibrotic in the control group. Thus, I-PRF appears to be a suitable biomaterial for enhancing bone regeneration in extraction sockets. Differences in the type of connective tissue between the two groups might indicate the optimal efficacy of I-PRF for enhancing osteogenesis.

Clark et al. [33] reported significantly higher mean osteogenesis in the A-PRF group than in the FDBA group. The new bone formation percentages in the present study in both groups were similar to other studies following the use of mineralized allografts for ridge preservation, i.e., 27%–68% [40, 41]. Moreover, Yoon, Lee, and Yoon [42] evaluated the effects of PRF on angiogenesis and osteogenesis in the guided bone regeneration (GBR) of cranial defects in rabbits. Although PRF increased the bone marrow cells, there was no significant difference between the intervention and control groups histomorphometrically and immunohistochemically. They used bovine bone minerals with/without PRF to regenerate cranial bone defects in rabbits.

Anwandter et al. [43] evaluated alveolar ridge dimension changes after tooth extraction and application of leukocyte- and platelet rich fibrin (L-PRF), reporting that L-PRF could enhance a positive clinical outcome in ridge preservation concerning the reduction of horizontal and vertical bone resorption. Temmerman et al. [44] evaluated the efficacy of L-PRF for ridge preservation. They found significantly greater bone fill (visible mineralized bone) in the intervention group, coinciding with the present results, although I-PRF was used along with allograft in the present study. In general, variations in surgical protocols used for ridge preservation can affect the outcomes. For instance, atraumatic methods bring about more favorable results than traumatic methods. Also, applying different collagen membranes affects the primary stability of the wound and, subsequently, the outcomes of ridge perseveration [45, 46].

No significant difference was found regarding inflammation and foreign body reactions between the two groups, possibly due to the short duration of the study. More histological changes might be observed over longer periods.

The present study showed a significantly higher percentage of residual graft material in the control group than in the I-PRF group. Allograft material appears to have a slower rate of resorption and remains in the extraction socket for some time. On the other hand, residual particles can preserve the ridge for implant placement [36, 45, 47, 48]. Nonetheless, optimal healing requires angiogenesis and new tissue regeneration in the wound, and allograft material may delay the healing process.

According to Mehta et al. [49], platelet-rich plasma (PRP) used as an adjunct with a xenograft resulted in a significant reduction in pocket depth and greater clinical attachment level gain in comparison to xenograft alone in patients with periodontitis and intrabony defects. Similar to our study, they used contained bony defects, but unlike our study, they used xenograft particles and PRP as we used PRF and allograft. The main limitation of this study was similar to our study and it was short following time of the patients.

Tallarico et al. [50] concluded that socket preservation with L-PRF mixed with decellularized bovine compact bone demonstrated favorable results compared with decellularized bovine compact bone from bovine femur alone. In our study, we used I-PRF with different preparations and allografts instead of L-PRF and xenograft in spite of these conclusions. Further studies with larger sample sizes and longer follow-up periods are needed in both studies to confirm these preliminary results.

Kadam et al. [51], in a study in 2023, aimed to explore the clinical and radiographic effectiveness of amoxicillin-incorporated PRF in the management of intrabony defects. Both groups seem to be effective in the treatment of intrabony defects despite the initial wound healing was superior in the test group. In this study, intrabony defects were assessed like ours; however, PRF clot was used instead of I-PRF. The main limitation of Kadam's study was the number of samples and the type of study like our study's limitations.

In total, the current findings have unveiled that the utilization of allograft in isolation has also demonstrated success in the preservation of ridges, albeit with inferior outcomes compared to the application of allograft in conjunction with I-PRF. The strength of the present study lies in its histological evaluation of human biopsy samples, a facet often absent in prior studies that predominantly relied on histological assessments conducted on animal models. A restricted duration of 3 months for the follow-up was a constraint in this investigation. Additional investigations with extended follow-up periods are necessary to evaluate the consequences of ridge preservation with I-PRF over a longer time frame. Detailed evaluation of periodontal status by biomarkers like sclerostin [52], etc., could be suggested for patients with better periodontal screening status in teeth next to extraction sites. Furthermore, it is advised that forthcoming studies employ a split-mouth design to eradicate the confounding influence of inter-individual variations on the healing process. Sample size calculation, socket situation before extraction, gingival biotype, and underestimation of the results because of sample size were the other limitations.

## 5. Conclusion and Recommendation

The present results revealed that allograft alone was also successful in ridge preservation, but the results were inferior to allograft + I-PRF. Histological assessment of human biopsy samples was a strength of the present study since most relevant previous studies performed histological assessments on animal models. A limited follow-up period of 3 months was a limitation of this study. Further studies with longer follow-ups are required to assess the outcome of ridge preservation with I-PRF over a longer period. Also, future studies with a split-mouth design are recommended to eliminate the confounding effect of inter-individual differences on healing. Despite the optimal efficacy of ridge preservation in both groups, the application of allograft plus I-PRF yielded superior histological, clinical, and radiographic results compared with allograft alone, and this difference was significant in most variables.

## Figures and Tables

**Table 1:** Measures of central dispersion for the variables measured in the two groups.

Variable measures	Group	S.E^b^	Mean (+/−SD)	CI (confidence interval)	Minimum	Maximum	Range	*P*-value^a^
Reduction in radiographic bone width	Control intervention	0.12 0.12	1.07 (0.40) 0.67 (0.39)	[0.38, 0.95] [0.78, 1.36]	0.65 0.13	1.91 1.37	1.26 1.24	0.038
Reduction in radiographic bone height	Control intervention	0.14 0.10	1.20 (0.46) 0.96 (0.34)	[0.86, 1.53] [0.71, 1.21]	0.68 0.56	2.12 1.68	1.44 1.12	0.213
Reduction in clinical bone width	Control intervention	0.17 0.12	1.31 (0.55) 0.82 (0.39)	[0.92, 1.71] [0.54, 1.10]	0.78 0.43	2.63 1.54	1.85 1.11	0.033
Percentage of osteogenesis	Control intervention	4.13 6.11	44.15% (13.09%) 60.72% (19.32%)	[34.79, 53.52] [46.89, 74.54]	21.02% 31.60%	61% 86.54%	39.98% 54.94%	0.038
Percentage of residual graft particles	Control intervention	3.23 2.57	30.75% (10.24%) 20.28% (8.14%)	[23.42, 38.08] [14.45, 26.10]	13.21% 8.03%	46.10% 37.48%	32.89% 29.45%	0.021
Percentage of nonmineralized tissue	Control intervention	3.40 3.10	25.76% (10.77%) 19.73% (9.82%)	[18.05, 33.46] [12.71, 26.76]	12% 8.03%	48.40% 42.10%	36.40% 34.07%	0.208

^a^Student's *t*-test.

^b^Standard error.

## Data Availability

The data used to support the findings of this study were supplied by the corresponding author under license, and data will be available upon reasonable request. Requests for access to these data should be made to the corresponding author.

## References

[1] Artzi Z., Tal H., Dayan D. (2000). Porous Bovine Bone Mineral in Healing of Human Extraction Sockets. Part 1: Histomorphometric Evaluations at 9 Months. *Journal of Periodontology*.

[2] Cicciù M., Cervino G., Fiorillo L. (2022). The Third Teething: Gerodontology and New Therapy Approaches. *Minerva Dental and Oral Science*.

[3] Tan W. L., Wong T. L., Wong M. C., Lang N. P. (2012). A Systematic Review of Post-Extractional Alveolar Hard and Soft Tissue Dimensional Changes in Humans. *Clinical Oral Implants Research*.

[4] Schropp L., Wenzel A., Kostopoulos L., Karring T. (2003). Bone Healing and Soft Tissue Contour Changes Following Single-Tooth Extraction: A Clinical and Radiographic 12-Month Prospective Study. *International Journal of Periodontics & Restorative Dentistry*.

[5] Ten Heggeler J. M., Slot D. E., Van der Weijden G. (2011). Effect of Socket Preservation Therapies Following Tooth Extraction in Non-Molar Regions in Humans: A Systematic Review. *Clinical Oral Implants Research*.

[6] Araújo M. G., Lindhe J. (2005). Dimensional Ridge Alterations following Tooth Extraction. An Experimental Study in the Dog. *Journal of Clinical Periodontology*.

[7] Zarb G. A., Schmitt A. (1990). the Longitudinal Clinical Effectiveness of Osseointegrated Dental Implants: The Toronto Study. Part II: The Prosthetic Results. *The Journal of Prosthetic Dentistry*.

[8] Thakur S., Martande S., Ankit K. (2023). Comparing the Effectiveness of Conventional and Microsurgical Access Flap Techniques in Managing Horizontal Bony Defects in Chronic Periodontitis Patients: A Clinical and Radiographic Study. *European Journal of General Dentistry*.

[9] Cicciù M., Fiorillo L., Cervino G., Habal M. B. (2021). Bone Morophogenetic Protein Application as Grafting Materials for Bone Regeneration in Craniofacial Surgery: Current Application and Future Directions. *Journal of Craniofacial Surgery*.

[10] Jambhekar S., Kernen F., Bidra A. S. (2015). Clinical and Histologic Outcomes of Socket Grafting After Flapless Tooth Extraction: A Systematic Review of Randomized Controlled Clinical Trials. *The Journal of Prosthetic Dentistry*.

[11] Iasella J. M., Greenwell H., Miller R. L. (2003). Ridge Preservation With Freeze-Dried Bone Allograft and a Collagen Membrane Compared to Extraction Alone for Implant Site Development: A Clinical and Histologic Study in Humans. *Journal of Periodontology*.

[12] Zitzmann N. U., Schärer P., Marinello C. (2001). Long-Term Results of Implants Treated With Guided Bone Regeneration: A 5-Year Prospective Study. *The International Journal of Oral & Maxillofacial Implants*.

[13] Guruprasad M., Kulloli A., Mehta V., Fiorillo L., Cicciu M. (2023). Maxillary Sinus Floor Augmentation With Allograft Alone Compared With Alternate Grafting Materials: A Systematic Review and Meta-Analysis. *The Journal of Craniofacial Surgery*.

[14] Choukroun J., Adda F., Schoeffer C., Vervelle A. (2000). PRF: An Opportunity in Perio-Implantology. *Implantodontie*.

[15] Hoaglin D. R., Lines G. K. (2013). Prevention of Localized Osteitis in Mandibular Third-Molar Sites Using Platelet-Rich Fibrin. *International Journal of Dentistry*.

[16] Aroca S., Keglevich T., Barbieri B., Gera I., Etienne D. (2009). Clinical Evaluation of a Modified Coronally Advanced Flap Alone or in Combination With a Platelet-Rich Fibrin Membrane for the Treatment of Adjacent Multiple Gingival Recessions: A 6-Month Study. *Journal of Periodontology*.

[17] Padma R., Shilpa A., Kumar P. A., Nagasri M., Kumar C., Sreedhar A. (2013). A Split Mouth Randomized Controlled Study to Evaluate the Adjunctive Effect of Platelet-Rich Fibrin to Coronally Advanced Flap in Miller’s Class-I and II Recession Defects. *Journal of Indian Society of Periodontology*.

[18] Agarwal S. K., Jhingran R., Bains V. K., Srivastava R., Madan R., Rizvi I. (2016). Patient-Centered Evaluation of Microsurgical Management of Gingival Recession Using Coronally Advanced Flap With Platelet-Rich Fibrin or Amnion Membrane: A Comparative Analysis. *European Journal of Dentistry*.

[19] Miranda M., Gianfreda F., Rosa A. (2023). Treatment of Oral Mucositis Using Platelet-Rich-Fibrin: A Retrospective Study on Oncological Patients. *Journal of Craniofacial Surgery*.

[20] Rosa A., Ranieri N., Miranda M., Mehta V., Fiorillo L., Cervino G. (2024). Mini Crestal Sinus Lift With Bone Grafting and Simultaneous Insertion of Implants in Severe Maxillary Conditions as an Alternative to Lateral Sinus Lift: Multicase Study Report of Different Techniques. *Journal of Craniofacial Surgery*.

[21] Dohan D. M., Choukroun J., Diss A. (2006). Platelet-Rich Fibrin (PRF): A Second-Generation Platelet Concentrate. Part III: Leucocyte Activation: A New Feature for Platelet Concentrates?. *Oral Surgery, Oral Medicine, Oral Pathology, Oral Radiology, and Endodontology*.

[22] Dohan D. M., Choukroun J., Diss A. (2006). Platelet-Rich Fibrin (PRF): A Second-Generation Platelet Concentrate. Part II: Platelet-Related Biologic Features. *Oral Surgery, Oral Medicine, Oral Pathology, Oral Radiology, and Endodontology*.

[23] Dohan D. M., Choukroun J., Diss A. (2006). Platelet-Rich Fibrin (PRF): A Second-Generation Platelet Concentrate. Part I: Technological Concepts and Evolution. *Oral Surgery, Oral Medicine, Oral Pathology, Oral Radiology, and Endodontology*.

[24] Fiorillo L., Cervino G., Galindo-Moreno P., Herford A. S., Spagnuolo G., Cicciù M. (2021). Growth Factors in Oral Tissue Engineering: New Perspectives and Current Therapeutic Options. *BioMed Research International*.

[25] Ghanaati S., Booms P., Orlowska A. (2014). Advanced Platelet-Rich Fibrin: A New Concept for Cell-Based Tissue Engineering by Means of Inflammatory Cells. *Journal of Oral Implantology*.

[26] Fujioka-Kobayashi M., Miron R. J., Hernandez M., Kandalam U., Zhang Y., Choukroun J. (2017). Optimized Platelet-Rich Fibrin With the Low-Speed Concept: Growth Factor Release, Biocompatibility, and Cellular Response. *Journal of Periodontology*.

[27] Dohan D. M., Choukroun J. (2007). PRP, cPRP, PRF, PRG, PRGF, FC … How to Find Your Way in the Jungle Of Platelet Concentrates?. *Oral Surgery, Oral Medicine, Oral Pathology, Oral Radiology, and Endodontology*.

[28] Shah R., Gowda T. M., Thomas R., Kumar T., Mehta D. S. (2019). Biological Activation of Bone Grafts Using Injectable Platelet-Rich Fibrin. *The Journal of Prosthetic Dentistry*.

[29] Alrayyes Y., Aloraini S., Alshagroud R., Binrayes A., Aljasse R. (2023). Extraction and Socket Preservation Before Implant Placement Using Freeze-Dried Allograft (FDBA) and Platelet-Rich Fibrin in Smokers: Radiographic and Histological Evaluation. *Applied Sciences*.

[30] Kawase T., Okuda K., Wolff L. F., Yoshie H. (2003). Platelet-Rich Plasma-Derived Fibrin Clot Formation Stimulates Collagen Synthesis in Periodontal Ligament and Osteoblastic Cells in Vitro. *Journal of Periodontology*.

[31] Mosesson M. (2005). Fibrinogen and Fibrin Structure and Functions. *Journal of Thrombosis and Haemostasis*.

[32] He L., Lin Y., Hu X., Zhang Y., Wu H. (2009). A Comparative Study of Platelet-Rich Fibrin (PRF) and Platelet-Rich Plasma (PRP) on the Effect of Proliferation and Differentiation of Rat Osteoblasts in Vitro. *Oral Surgery, Oral Medicine, Oral Pathology, Oral Radiology, and Endodontology*.

[33] Clark D., Rajendran Y., Paydar S. (2018). Advanced Platelet-Rich Fibrin and Freeze-Dried Bone Allograft for Ridge Preservation: A Randomized Controlled Clinical Trial. *Journal of Periodontology*.

[34] Thakkar D. J., Deshpande N. C., Dave D. H., Narayankar S. D. (2016). A Comparative Evaluation of Extraction Socket Preservation With Demineralized Freeze-Dried Bone Allograft Alone and Along With Platelet-Rich Fibrin: A Clinical and Radiographic Study. *Contemporary Clinical Dentistry*.

[35] Hauser F., Gaydarov N., Badoud I., Vazquez L., Bernard J.-P., Ammann P. (2013). Clinical and Histological Evaluation of Postextraction Platelet-Rich Fibrin Socket Filling: A Prospective Randomized Controlled Study. *Implant Dentistry*.

[36] Avila-Ortiz G., Elangovan S., Kramer K. W. O., Blanchette D., Dawson D. V. (2014). Effect of Alveolar Ridge Preservation After Tooth Extraction: A Systematic Review and Meta-Analysis. *Journal of Dental Research*.

[37] Girish Kumar N., Chaudhary R., Kumar I., Arora S. S., Kumar N., Singh H. (2018). To Assess the Efficacy of Socket Plug Technique Using Platelet Rich Fibrin With or Without the use of Bone Substitute in Alveolar Ridge Preservation: A Prospective Randomised Controlled Study. *Oral and Maxillofacial Surgery*.

[38] Zhang Y., Ruan Z., Shen M. (2018). Clinical Effect of Platelet-Rich Fibrin on the Preservation of the Alveolar Ridge Following Tooth Extraction. *Experimental and Therapeutic Medicine*.

[39] Suttapreyasri S., Leepong N. (2013). Influence of Platelet-Rich Fibrin on Alveolar Ridge Preservation. *Journal of Craniofacial Surgery*.

[40] Beck T. M., Mealey B. L. (2010). Histologic Analysis of Healing After Tooth Extraction With Ridge Preservation Using Mineralized Human Bone Allograft. *Journal of Periodontology*.

[41] Fotek P. D., Neiva R. F., Wang H. (2009). Comparison of Dermal Matrix and Polytetrafluoroethylene Membrane for Socket Bone Augmentation: A Clinical and Histologic Study. *Journal of Periodontology*.

[42] Yoon J.-S., Lee S.-H., Yoon H.-J. (2014). the Influence of Platelet-Rich Fibrin on Angiogenesis in Guided Bone Regeneration Using Xenogenic Bone Substitutes: A Study of Rabbit Cranial Defects. *Journal of Cranio-Maxillofacial Surgery*.

[43] Anwandter A., Bohmann S., Nally M., Castro A. B., Quirynen M., Pinto N. (2016). Dimensional Changes of the Post Extraction Alveolar Ridge, Preserved With Leukocyte- and Platelet Rich Fibrin: A Clinical Pilot Study. *Journal of Dentistry*.

[44] Temmerman A., Vandessel J., Castro A. (2016). the use of Leucocyte and Platelet-Rich Fibrin in Socket Management and Ridge Preservation: A Split-Mouth, Randomized, Controlled Clinical Trial. *Journal of Clinical Periodontology*.

[45] Becker W., Clokie C., Sennerby L., Urist M. R., Becker B. E. (1998). Histologic Findings After Implantation and Evaluation of Different Grafting Materials and Titanium Micro Screws Into Extraction Sockets: Case Reports. *Journal of Periodontology*.

[46] Brownfield L. A., Weltman R. L. (2012). Ridge Preservation With or Without an Osteoinductive Allograft: A Clinical, Radiographic, Micro-Computed Tomography, and Histologic Study Evaluating Dimensional Changes and New Bone Formation of the Alveolar Ridge. *Journal of Periodontology*.

[47] Arunyanak S. P., Pollini A., Ntounis A., Morton D. (2017). Clinician Assessments and Patient Perspectives of Single-Tooth Implant Restorations in the Esthetic Zone of the Maxilla: A Systematic Review. *The Journal of Prosthetic Dentistry*.

[48] Carmagnola D., Adriaens P., Berglundh T. (2003). Healing of Human Extraction Sockets Filled With Bio-Oss®. *Clinical Oral Implants Research*.

[49] Mehta V., Fiorillo L., Langaliya A., Obulareddy V. T., Cicciu M. (2023). The Effect of Xenograft and Platelet-Rich Plasma in the Surgical Management of Intrabony Defects in Periodontitis Patients: A Systematic Review. *Journal of Craniofacial Surgery*.

[50] Tallarico M., Xhanari E., Lumbau A. M. (2022). Histological and Histomorphometric Evaluation of Post-Extractive Sites Filled With a New Bone Substitute With or Without Autologous Plate Concentrates: One-Year Randomized Controlled Trial. *Materials*.

[51] Kadam S., Kulloli A., Shetty S. K. (2023). Comparative Evaluation of Efficacy of Antibiotics Incorporated Platelet Rich Fibrin Versus Platelet Rich Fibrin Alone in the Treatment of Intrabony Defects. *Journal of Population Therapeutics and Clinical Pharmacology*.

[52] Mathur A., Gopalakrishnan D., Sharath S., Mehta V., Bagwe S., Kumbhalwar A. (2024). Comparative Evaluation of Sclerostin Levels in Gingival Crevicular Fluid of Periodontal Health and Disease Before and After Nonsurgical Periodontal Therapy. *Journal of Datta Meghe Institute of Medical Sciences University*.

